# Supplementary material for: Selective Copolymerization from Mixed Monomers of Phthalic Anhydride, Propylene Oxide and Lactide Using Nano-Sized Zinc Glutarate
Source: Nanomaterials (Basel). 2024 Sep 22;14(18):1535. doi: 10.3390/nano14181535 (PMC11434771; doi:10.3390/nano14181535)
Supplement: Supplementary file 1 [file nanomaterials-14-01535-s001.zip › nanomaterials-3130327-supplementary.pdf]

Supporting Information

Selective Copolymerization from Mixed Monomers of Phthalic Anhydride, Propylene Oxide and Lactide Using Nano-Sized Zinc Glutarate

Xiaoting Zhang †, Zhidong Li †, Liyan Wang, Jingjing Yu, Yefan Liu and Pengfei Song *

College of Chemistry and Chemical Engineering, Key Laboratory of Eco-Functional Polymer Materials of the Ministry of Education, Key Laboratory of Eco-Environmental Polymer Materials of Gansu Province, Gansu International Scientific and Technological Cooperation Base of Water-Retention Chemical Functional Materials, Northwest Normal University, Lanzhou 730070, China

* Correspondence: songpf@nwnu.edu.cn

† These authors contributed equally to this work.

Table S1. X-ray data of ZnGA.

Peak No.	2θ°	FWHM	Intensity	Intensity/FWHM	*L*(Å)
a	12.70	0.376	916	2436	387.5
b	22.54	0.259	775	2992	570.1
c	23.00	0.353	762	2159	418.7

Table S2. The textural properties of ZnGA

Catalyst	SSA(m^2/g)[a]	Pore diameter(nm)[b]	Pore volume($\times 10^2 cm^3/g$)[b]
ZnGA	1.15	60.3	0.017

[a] The specific surface area (SSA) of the catalysts was obtained from nitrogen adsorption experiment and calculated by BET analysis.

[b] Pore size and pore volume were calculated by BJH analysis.

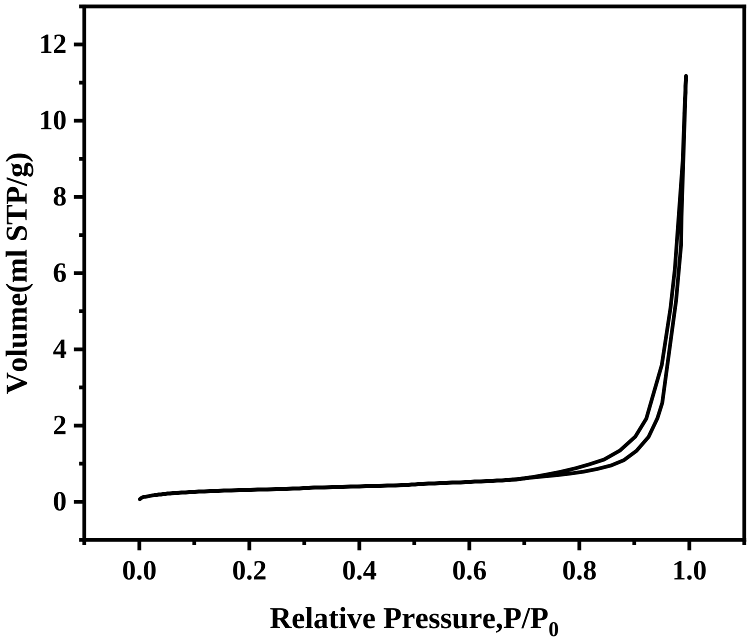

Figure S1. N_2 adsorption/desorption isotherms of ZnGA.

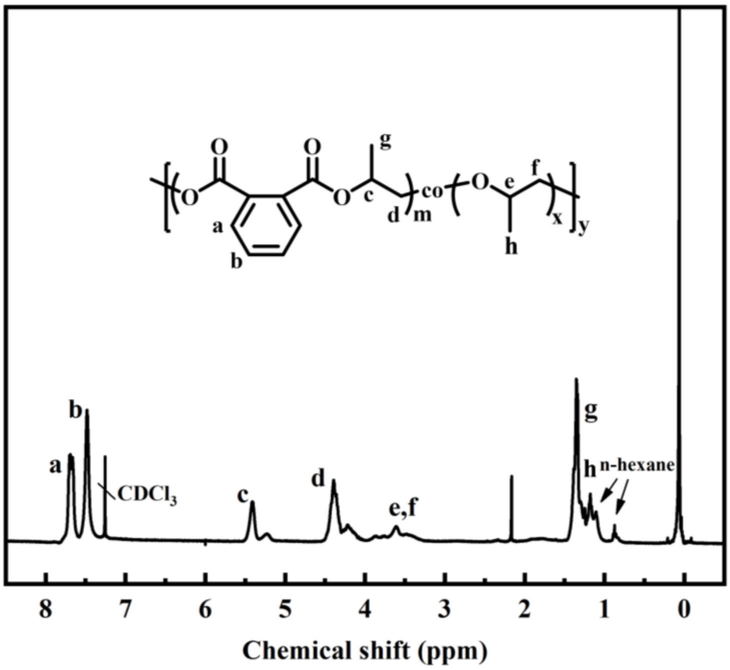

Figure S2. The ¹H NMR spectrum of PPAPO-co-PPO (Table 1, entry 5).

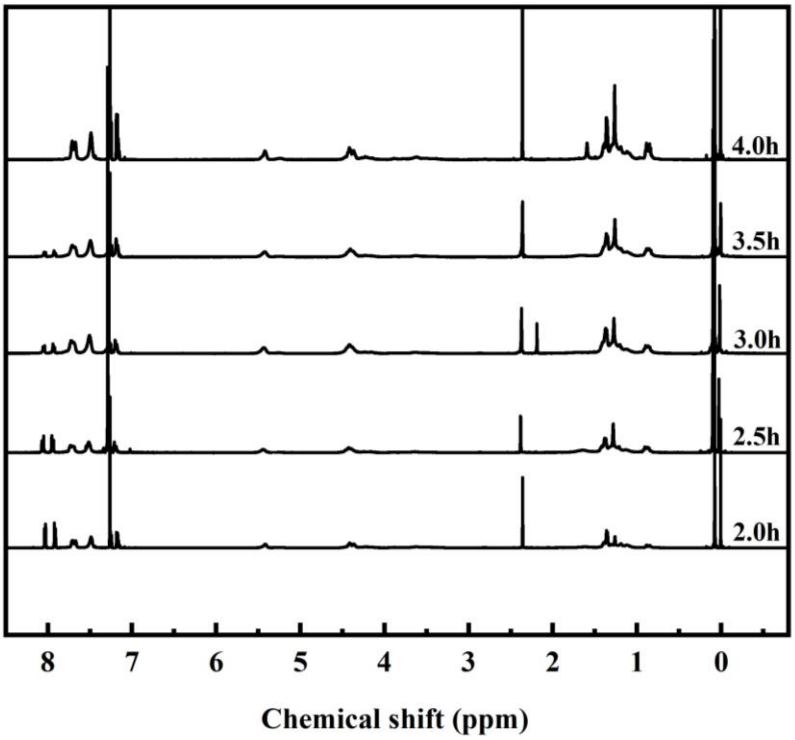

Figure S3. The ¹H NMR spectra of crude aliquots withdrawn from the reaction system of PA and PO copolymer.

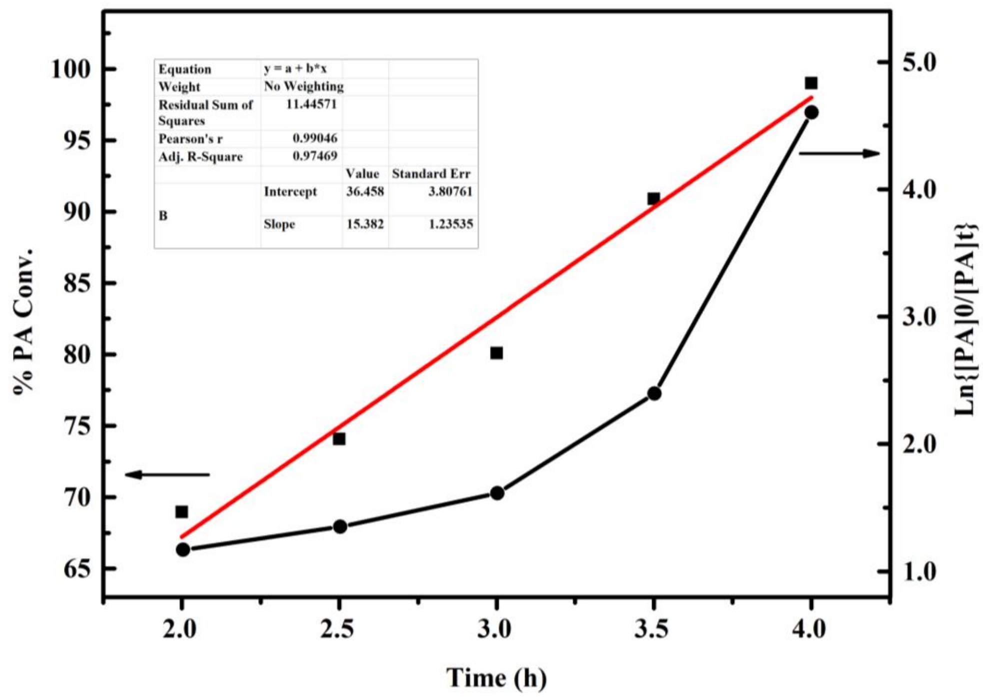

Figure S4. The fittings of zero-order kinetic plots and first-order kinetic plots with respect to PA.

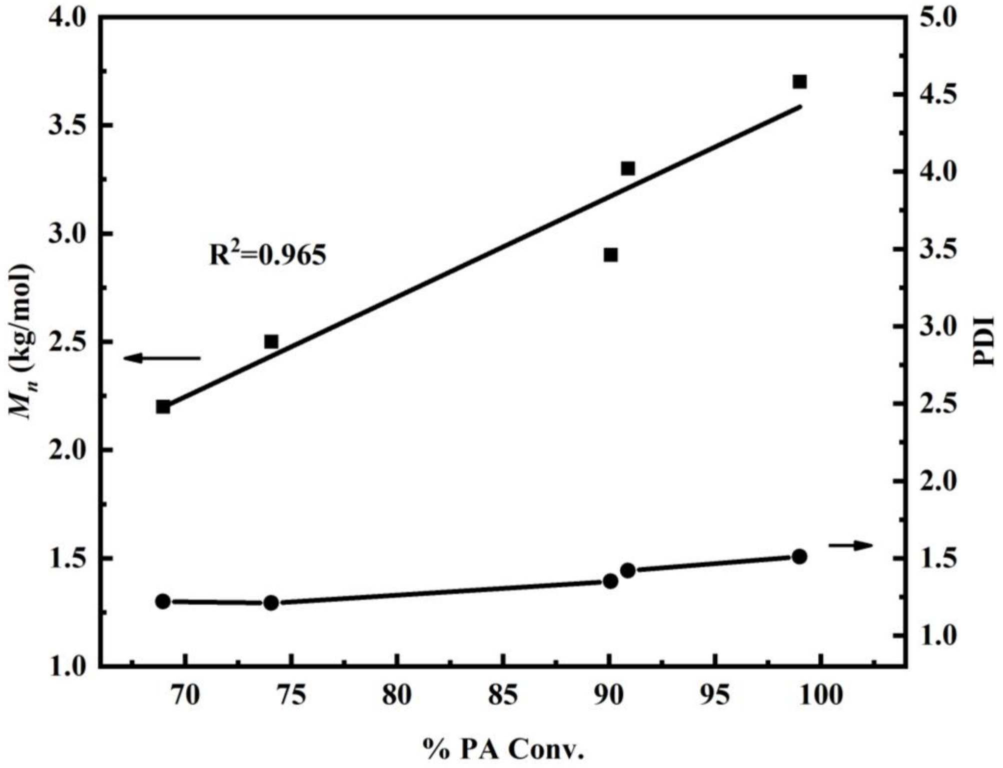

Figure S5. The plots of M_n and PDI versus PA conversion for the ROCOP of PA and PO.

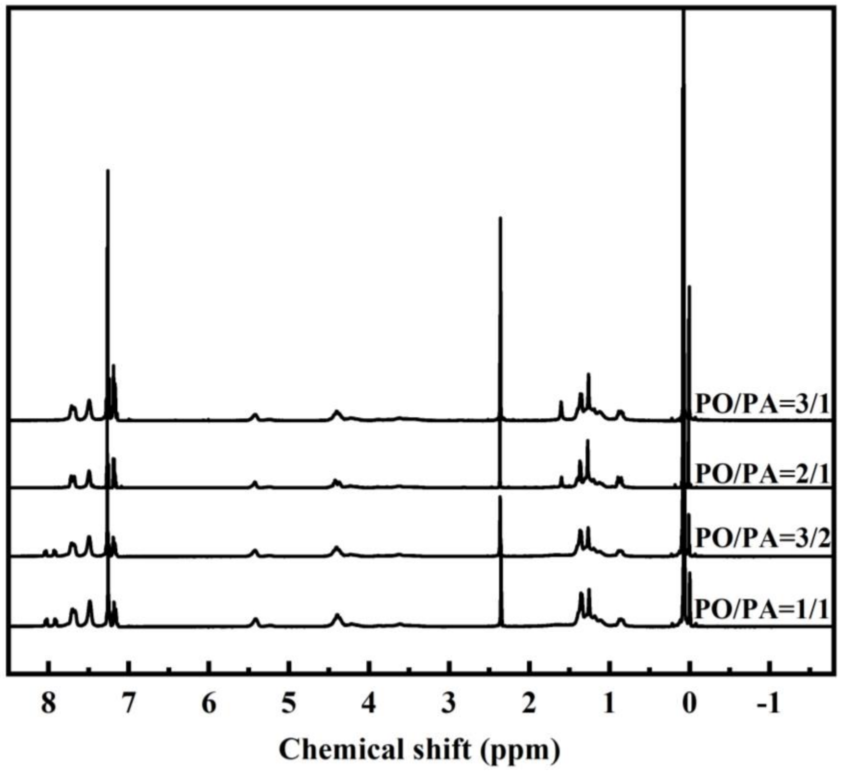

Figure S6. The ^{1}H NMR spectra of PPAPO-co-PPO at different molar feed ratios.

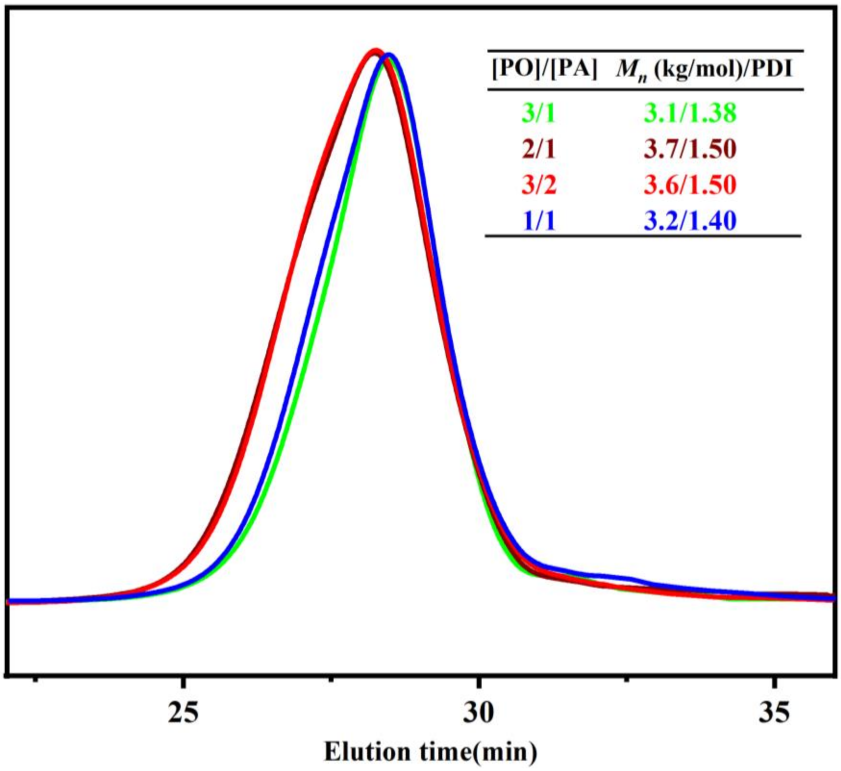

Figure S7. GPC traces of PPAPO-co-PPO at different molar feed ratios.

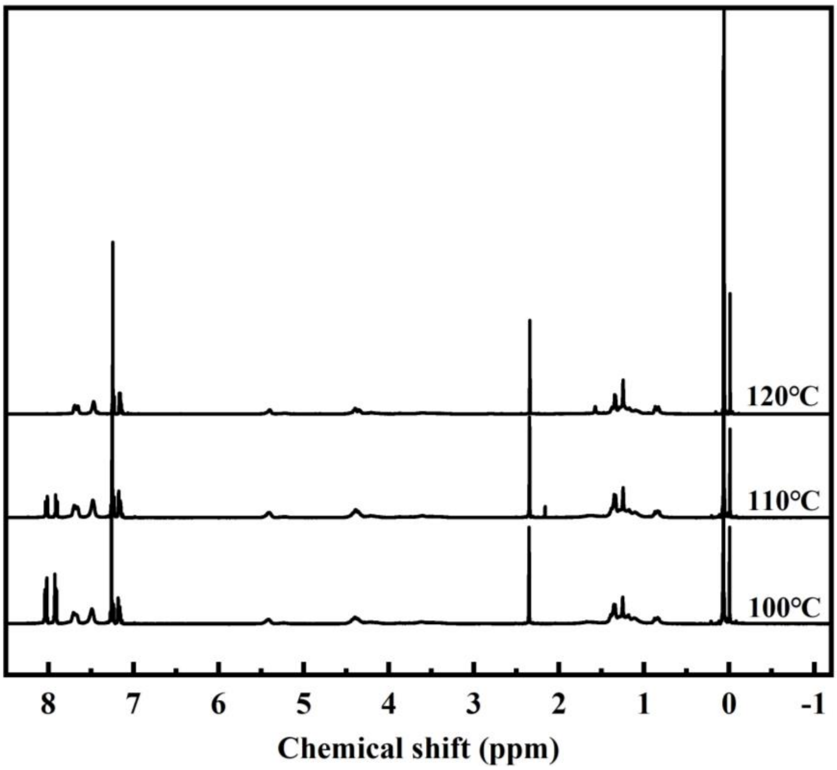

Figure S8. The ^{1}H NMR spectra of PPAPO-co-PPO at different temperatures.

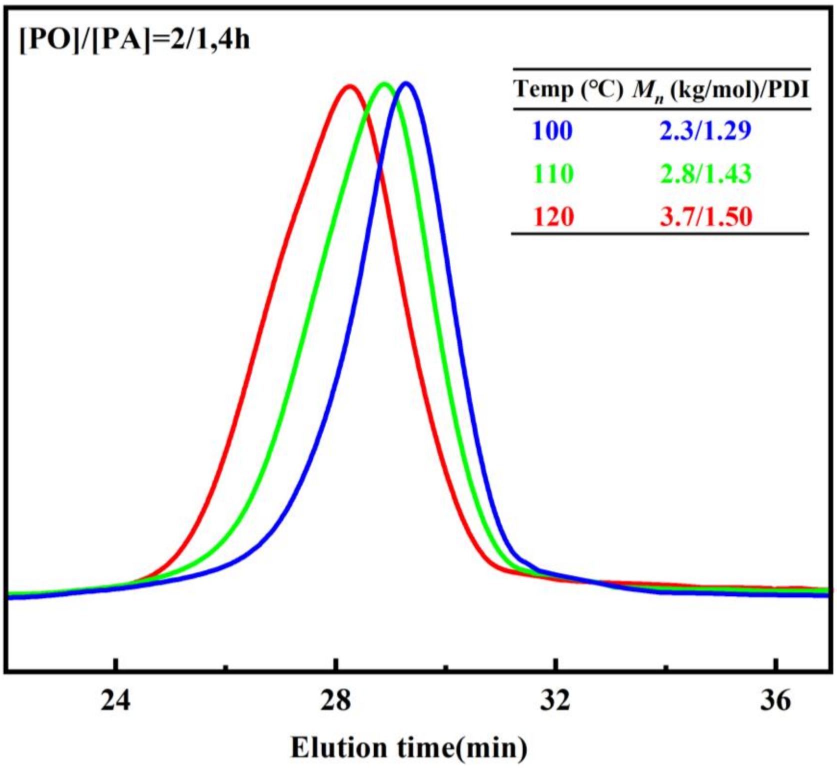

Figure S9. GPC traces of PPAPO-co-PPO at different temperatures.

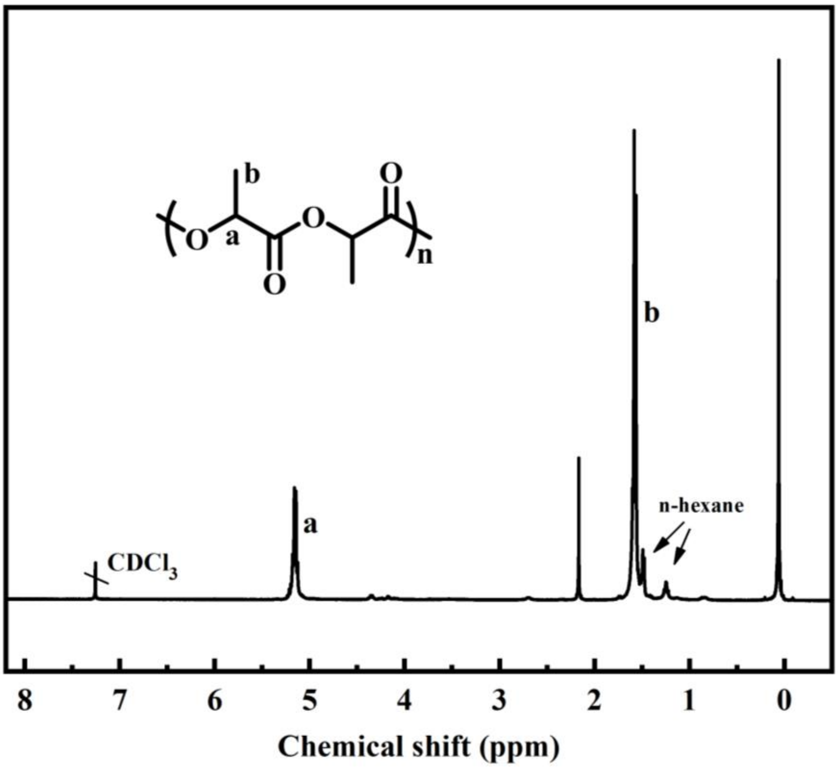

Figure S10. The ¹H NMR spectra of PLA (Table 2, entry 7).

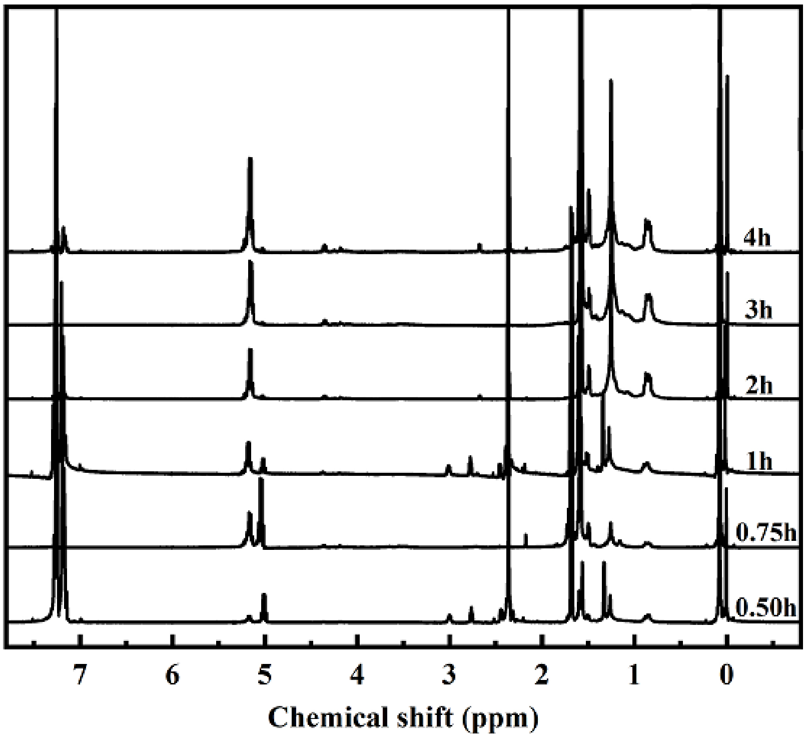

Figure S11. The ¹H NMR spectra of crude aliquots withdrawn from the reaction system of LA and PO copolymer.

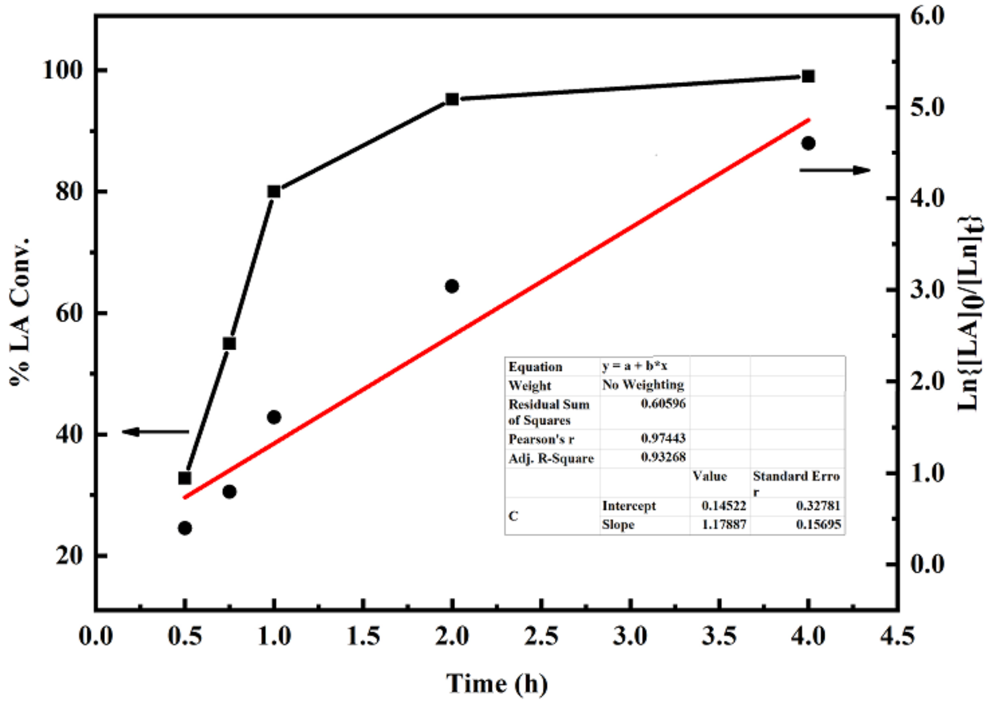

Figure S12. The fittings of zero-order kinetic plots and first-order kinetic plots with respect to LA.

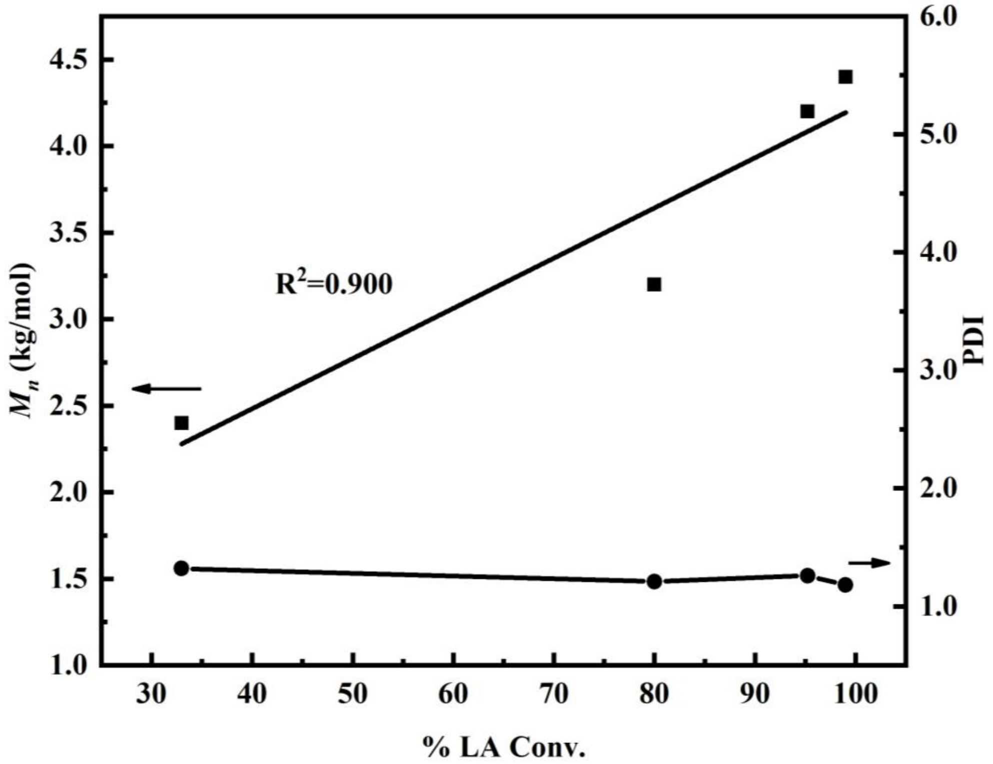

Figure S13. The plots of M_n and PDI versus LA conversion for the ROP of LA.

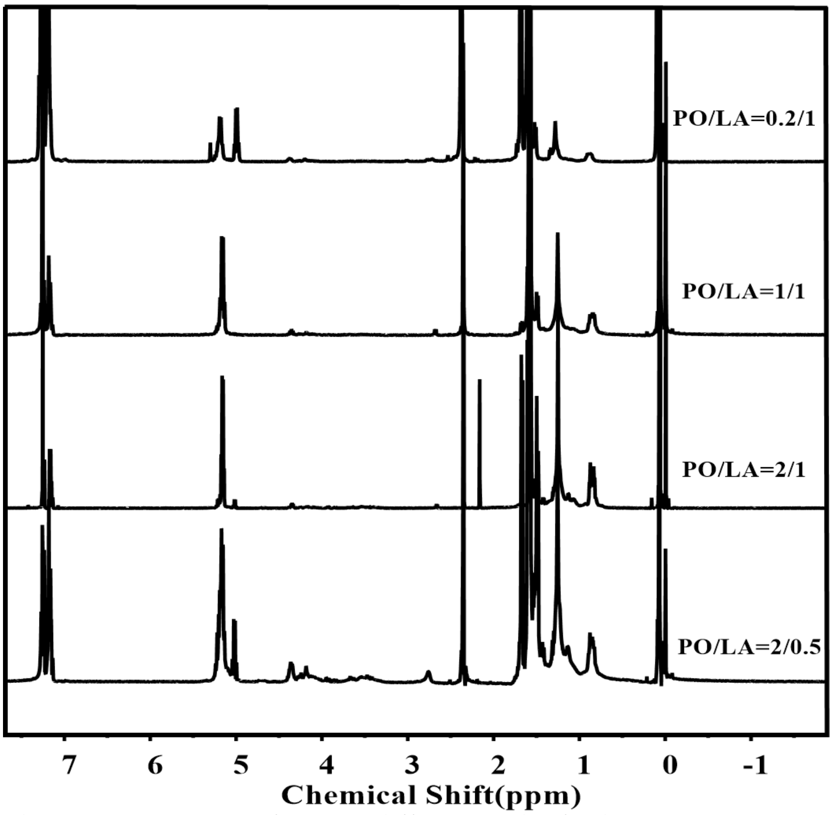

Figure S14. The ^{1}H NMR spectra of PLA at different molar feed ratios.

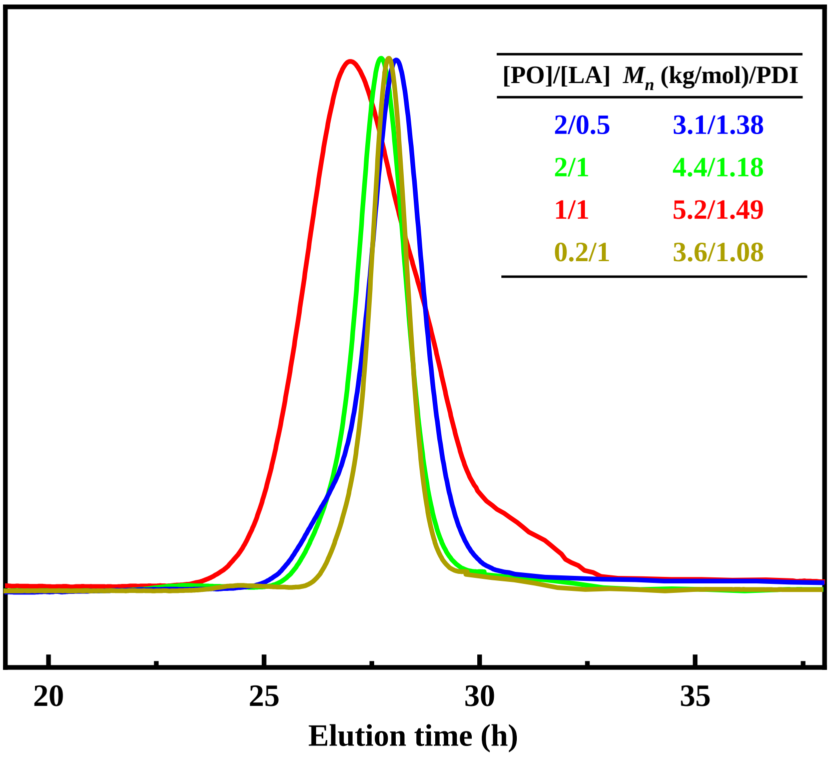

Figure S15. GPC traces of PLA at different molar feed ratios.

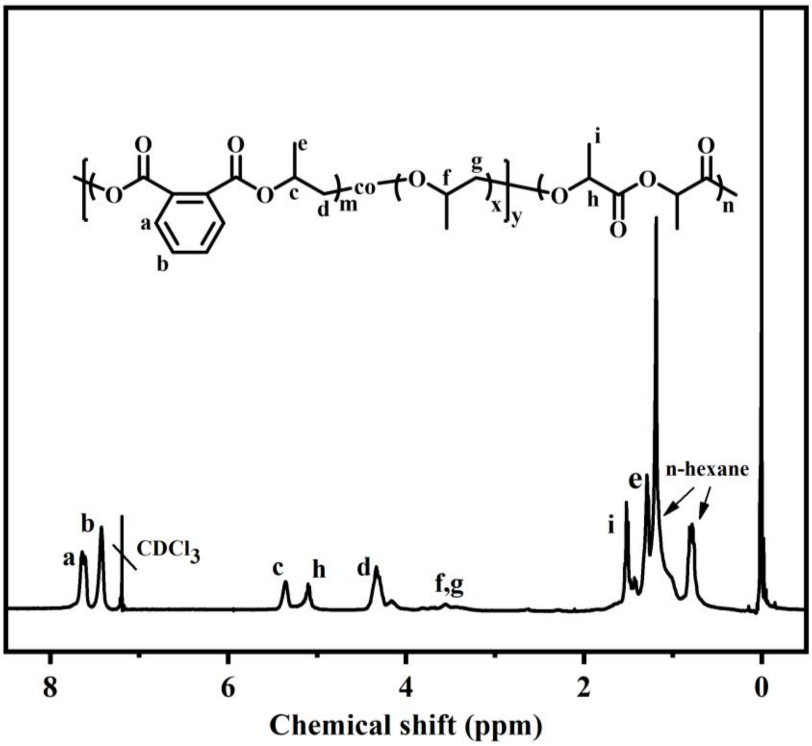

Figure S16. The ^{1}H NMR spectra of PA, PO and LA terpolymer (Table 3, entry 8).

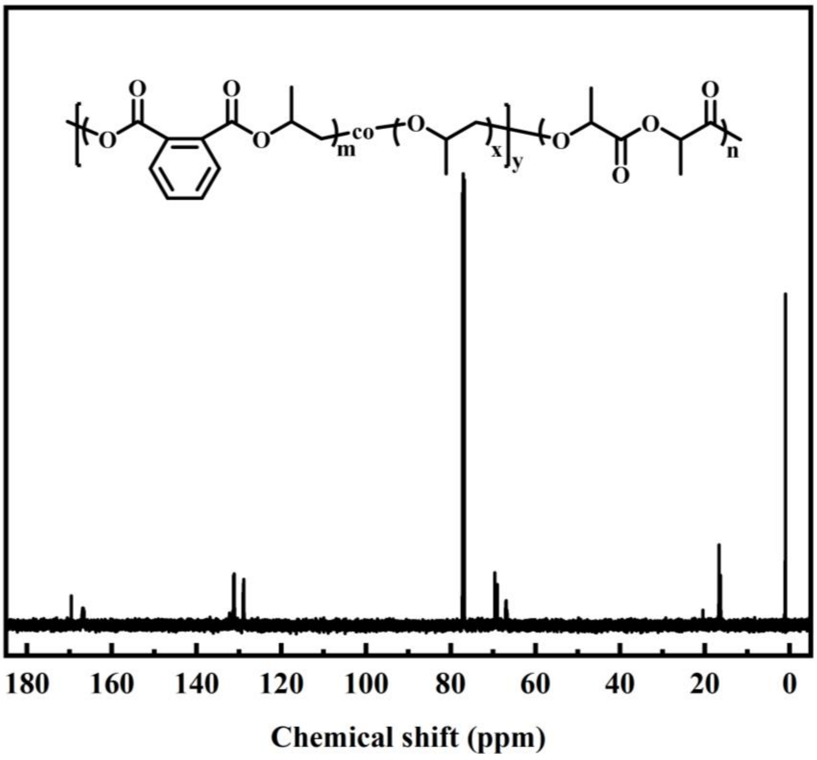

Figure S17. The ^{13}C NMR spectra of PA, PO and LA terpolymer (Table 3, entry 8).

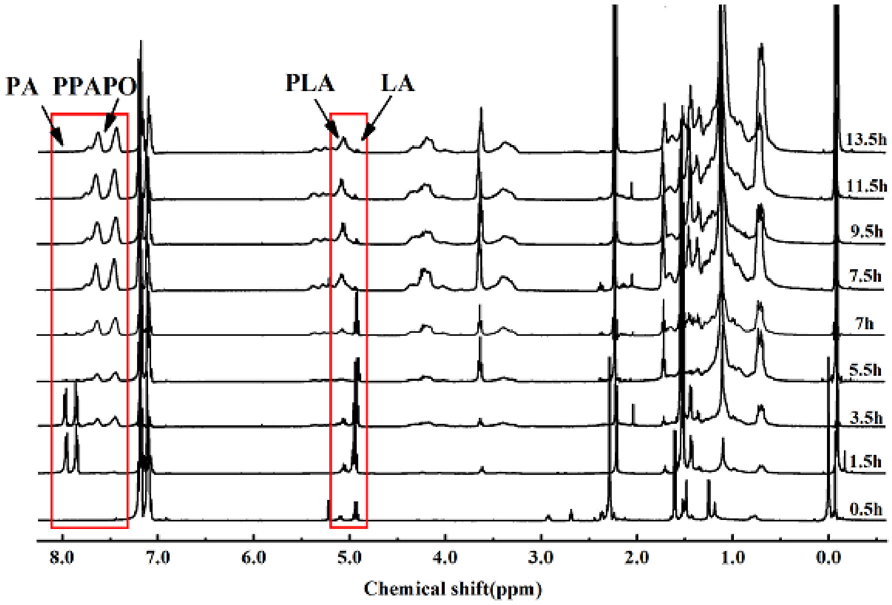

Figure S18. The ^{1}H NMR spectra in situ of crude aliquots withdrawn from the reaction system for monitoring the conversion of PA and LA and the formation of (PPAPO-co-PPO)-*b*-PLA. The copolymerization reactions were conducted in 5 ml toluene at 120 °C, 0.2 g ZnGA, [PA]:[LA]= 2/1; another 1 equiv of PA was added into the mixtures when the reaction was extended to 0.5 h.
